# Comparing dislocation rates by approach following elective primary dual mobility total hip arthroplasty: a systematic review

**DOI:** 10.1186/s13018-023-03724-6

**Published:** 2023-03-22

**Authors:** Justin T. Butler, Samuel D. Stegelmann, Johnathon L. Butler, Matthew Bullock, Richard M. Miller

**Affiliations:** 1 Department of Orthopedic Surgery, Mercy Health St. Vincent Medical Center, 2409 Cherry St, Suite #10, Toledo, OH 43608 USA; 2 Department of Orthopedic Surgery, Marshall University Joan C. Edwards School of Medicine, Huntington, WV USA

**Keywords:** Total hip arthroplasty, Total hip replacement, Osteoarthritis, Hip dislocation, Dual mobility

## Abstract

**Background:**

Dual mobility components can be implanted during total hip arthroplasty (THA) for primary osteoarthritis via a direct anterior approach (DAA), anterolateral approach (ALA), direct lateral approach (DLA), or posterior/posterolateral approach (PLA). This review compares dual mobility hip dislocation rates using these approaches for elective primary THA.

**Methods:**

PubMed, Embase, and Cochrane databases were systematically searched for articles published after January 1, 2006 that reported dislocation rates for adult patients after primary THA with dual mobility implants. Articles were excluded if they reported revision procedures, nonelective THA for femoral neck fractures, acetabular defects requiring supplemental implants, prior surgery, or ≤ 5 patients. The primary outcome was hip dislocation rate. Secondary outcomes included infection, Harris Hip Score (HHS), and Postel-Merle d’Aubigné (PMA) score.

**Results:**

After screening 542 articles, 63 met inclusion criteria. Due to study heterogeneity, we did not perform a meta-analysis. Eight studies reported DAA, 5 reported ALA, 6 reported the DLA, and 56 reported PLA. Study size ranged from 41 to 2,601 patients. Mean follow-up time ranged from 6 months to 25 years. Rates of infection and dislocation were low; 80% of ALA, 87.5% of DAA, 100% of DLA, and 82.1% of PLA studies reported zero postoperative dislocations. Studies reporting postoperative HHS and PMA scores showed considerable improvement for all approaches.

**Conclusions:**

Patients undergoing primary THA with dual mobility implants rarely experience postoperative dislocation, regardless of surgical approach. Additional studies directly comparing DAA, ALA, DLA, and PLA are needed to confirm these findings.

**Supplementary Information:**

The online version contains supplementary material available at 10.1186/s13018-023-03724-6.

## Background

Total hip arthroplasty (THA) is a common treatment for patients with hip osteoarthritis (OA). THA procedures have been projected to increase 174% from 2005 to 2030 in the United States [1]. Hip dislocation after THA is a leading cause of early surgical revision [2], and prevention of this complication is necessary to improve patient outcomes and reduce healthcare costs. Dual mobility components have been demonstrated to reduce rates of postoperative dislocation compared to standard cup implants in patients undergoing THA [3–5], although surgical approach during implantation may also affect clinical and functional outcomes [6–8].

The dual mobility cup concept was first developed in 1974 by Bousquet and Rambert [9]. A dual mobility construct has two points of articulation: the first interface is between small femoral head articulating within a larger mobile head, while the second interface is the larger head articulating with the acetabular component. This design has been demonstrated to increase the maximum hip range of motion prior to dislocation thus improving hip stability [10, 11]. Many studies have demonstrated the low dislocation rate utilizing dual mobility implants [12, 13]. Dual mobility has gained popularity for high-risk dislocation patients including those with revision surgery, osteonecrosis, hip dysplasia, femoral neck fracture, neuromuscular disorders, elderly, obesity, spinal fusions, and variability between functional spinopelvic relationship.

Dual mobility constructs may be implanted utilizing any surgical approach. The most common approaches include the direct anterior approach (DAA), anterolateral approach (ALA), direct lateral approach (DLA) or posterolateral approach (PLA). The DAA uses the natural intermuscular interval between the tensor fascia latae (TFL) and sartorius muscles [14]. The ALA begins farther laterally and uses the interval between the TFL and gluteus medius to expose the hip capsule [15]. The DLA begins laterally splitting the gluteus medius and vastus lateralis. The PLA does not follow a true interval to expose the hip posteriorly [16].

While the PLA has been the most commonly utilized approach, a major concern has been an increased risk for dislocation [17–19], especially without capsular and external rotator repair [20]. Studies have shown that the DAA, ALA, and DLA may lead to uniformly improved stability and earlier recovery versus the PLA [17, 21, 22], which has led to a more recent increase in utilization of dual mobility implants while performing the PLA, especially in high-risk patients [23].

At present, research directly comparing surgical approaches in patients receiving dual mobility implants is limited. This systematic review compares rates of dislocation by surgical approach in patients receiving dual mobility implants for elective primary THA.

## Methods

### Search Strategy

This study was performed in compliance with Preferred Items for Systematic Reviews and Meta-Analysis (PRISMA) [24]. PubMed, Embase, and Cochrane databases (or their application programming interface [API]) were used to identify potentially relevant studies published between January 2006 and January 2023. Nested Knowledge software (AutoLit; Nested Knowledge, St. Paul, MN, USA) was used to perform all searches, screening, and data extraction. The following search string was used: "total hip arthroplasty" AND "dual mobility" AND "dislocation". Additional studies were added based on expert recommendation. Two independent reviewers screened all titles and abstracts; remaining articles underwent full text screening. Any disagreements were resolved by consensus.

### Inclusion/exclusion criteria

For inclusion, articles had to describe rates of hip dislocation in adults (> 18 years of age) following elective primary THA with dual mobility implants via DAA, ALA, DLA, or PLA. Studies with multiple approaches were included if they stated the outcomes by approach. Articles were excluded for the following reasons: nonelective THA performed for femoral neck fracture; revision THA; periacetabular defects requiring supplemental implants; prior surgery; non-clinical study (in vitro*, *in vivo*, *in silico*,* animal, cadaver); case reports or case series with ≤ 5 patients; review or meta-analysis; editorial, letter, or commentary; abstract, protocol, or technical note; insufficient details on the intervention or outcomes; duplicate patient population; not relevant to the topic; not available in English; and full text unavailable. Study cohorts for primary THA indications were extracted and included when outcomes were clearly differentiated (i.e., excluding femoral neck fractures and revision THA).

### Risk of bias

The Joanna Briggs Institute forms for case series [25] and cohort studies [26] were used to assess risk of bias of the included studies. Individual questions pertaining to study design and execution were addressed with “yes,” “no,” “unclear,” or “not applicable” responses based on the quality of reporting in the manuscript and information from the authors. Two independent reviewers rated each question, while a third reviewer adjudicated discrepancies and rated studies as high, moderate, or low risk of bias.

### Data collection

Patient baseline characteristics were collected as available, including age, sex, and indication for THA (e.g., osteoarthritis). The primary outcome of interest was rate of dislocation. Secondary outcomes included infection, all-cause mortality, and hip function as assessed by the Harris Hip Score (HHS) [27] and Postel-Merle d’Aubigné (PMA) score [28]. Patient baseline characteristics and outcome scores were only presented when representative of the entire cohort included (i.e., no patients removed for excluded surgical indications).

### Statistical analysis

Baseline characteristics and outcomes are summarized using frequencies (*n*/*N*), percentages (%), mean ± standard deviation, median, and range, as appropriate. Due to heterogeneous study designs and patient populations, variable study quality, and incomplete reporting, meta-analyses were not performed, and no inferential statistics were summarized for this review.

## Results

### Search results

**Fig. 1:**
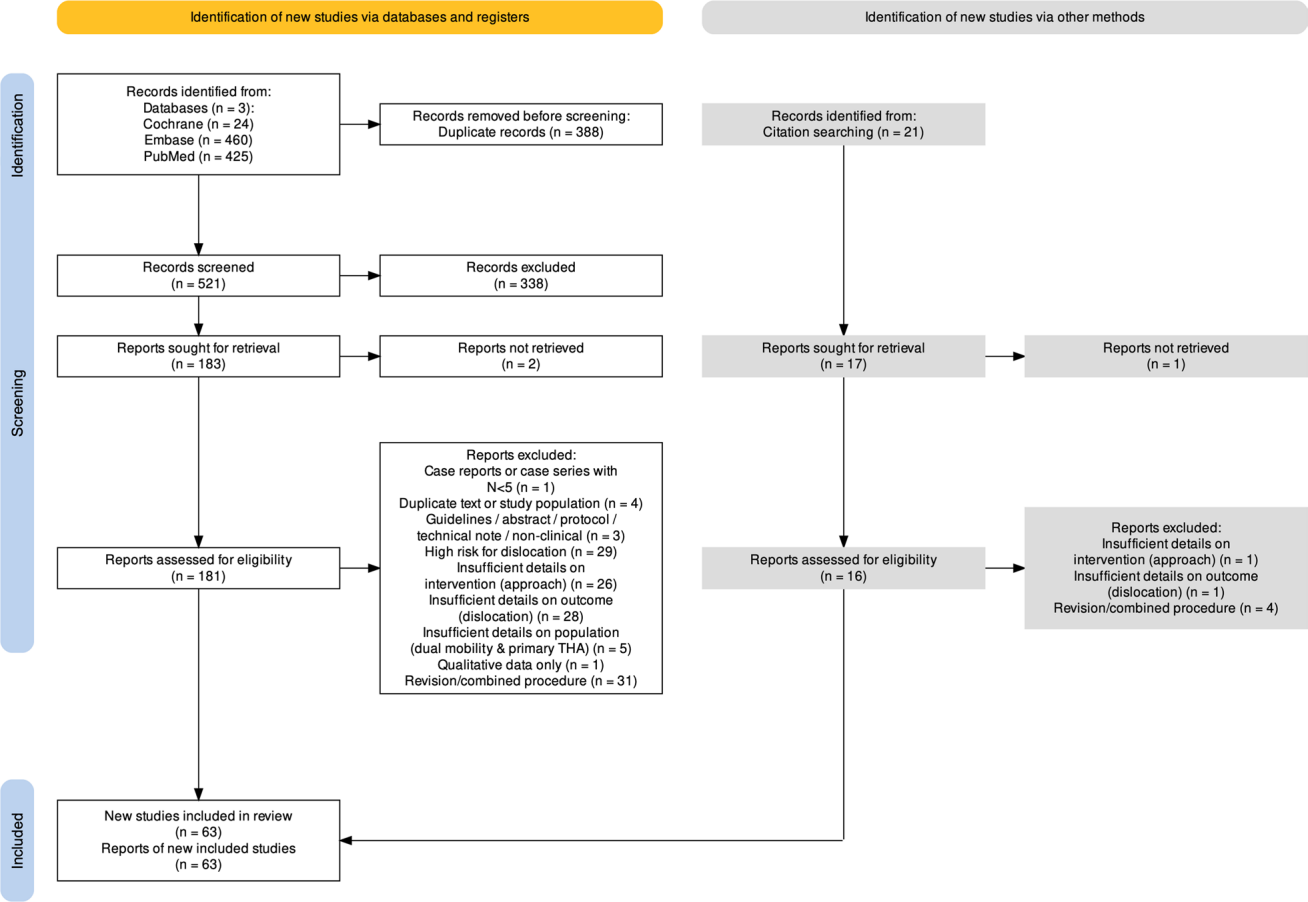
PRISMA diagram showing study attrition

A total of 521 records were identified using the search strategy, and 21 additional records were added by expert recommendation. After screening based on title and abstract, 197 articles were retrieved for full text review, and 63 articles met all inclusion criteria [2, 10, 29–90]. Full details of study attrition with exclusion reasons are shown in Fig. 1.

### Risk of bias

Full details of the risk of bias assessment are available in Additional file 1: Table S1. Four studies (6.3%) were considered high risk of bias, and 18 (28.6%) were considered moderate risk. The remaining 41 studies (65.1%) were deemed low risk of bias. All studies were ultimately recommended for inclusion.

### Study and patient characteristics

Of the 63 articles included, 11 reported outcomes for two different approaches in the same study [10, 33, 40, 43, 49, 50, 52, 69, 79, 84], and one study reported three approaches [74], resulting in 75 total study arms. Eight studies reported the DAA, five reported the ALA, six reported the DLA, and 56 studies reported the PLA (Table 1). Cohort size ranged from 6 to 2601 subjects [69, 70]. Some studies reported outcomes per number of operated hips while others reported per number of patients. In studies reporting an indication for surgery, the most common indications were osteoarthritis, osteonecrosis, hip dysplasia, and post-traumatic arthritis (Table 2).

**Table 1:** Study characteristics and demographics

Author	References	Nationality	LOE	Approach	Size (hips)	Age (years)	Sex (female)
*Anterolateral*							
Chughtai et al.	[41]	USA	IV	ALA	410	64 ± 12	246 (60.0)
Fessy et al.^†^	[50]	France	III	ALA	71	–	–
Lamo-Espinosa et al.	[64]	Spain	IV	ALA	68	81.7 ± 6.4	53 (77.9)
Vielpeau et al.	[87]	France	IV	ALA	668	65.7	301 (49.3)
*Direct Anterior*							
Baker et al.	[32]	USA	IV	DAA	152	–	–
Batailler et al.^†^	[33]	France	III	DAA	199	–	–
Homma et al.	[61]	Japan	III	DAA	81	76.2 ± 6.9	69 (85.2)
Randelli et al.	[80]	Italy	IV	DAA	26	69.3 ± 12.7	16 (61.5)
Singh et al.^†^	[84]	USA	III	DAA	55	61.2 ± 12.1	40 (72.7)
*Direct Lateral*							
Henawy and Badie	[59]	Egypt	IV	DLA	8	–	–
*Posterior*							
Acker et al.	[29]	Switzerland	III	PLA	15	–	–
Almeida	[30]	Brazil	IV	PLA	30	–	–
Asselineau et al.	[31]	France	IV	PLA	350	68	233 (61.9)
Assi et al.	[2]	Lebanon	IV	PLA	66	–	–
Batailler et al.^†^	[33]	France	III	PLA	97	–	–
Bauchu et al.	[34]	France	IV	PLA	150	69	89 (59.3)
Beckert et al.	[35]	USA	III	PLA	361	–	–
Belgaïd et al.	[36]	France	IV	PLA	128	83*	89 (69.5)
Bouchet et al.	[37]1	France	III	PLA	105	76.6 ± 5.7	60 (57.1)
Chalmers et al.	[38]	USA	IV	PLA	305	68	149 (48.9)
Chalmers et al.	[39]	USA	IV	PLA	86	69	57 (71.3)
Dagneaux et al.	[42]	France	III	PLA	41	77 ± 7	27 (64.3)
Dubin et al.	[44]	USA	III	PLA	574	67.9 ± 10.2	278 (48.4)
Dubin et al.	[45]	USA	IV	PLA	664	61.7 ± 9.2	308 (46.4)
Dubin et al.	[46]	USA	III	PLA	140	–	–
Epinette et al.	[47]	France	II	PLA	437	74.2	283 (64.8)
Epinette et al.	[48]	France	II	PLA	31	–	–
Fessy et al.^†^	[50]	France	III	PLA	469	–	–
Fiquet and Noyer	[51]	France	IV	PLA	450	–	–
Fresard et al.	[53]	France	IV	PLA	134	74 ± 6	75 (59.1)
Gaillard et al.	[54]	France	IV	PLA	138	68	73 (52.9)
Gkiatas et al.	[55]	USA	IV	PLA	205	79.3 ± 7.6	162 (79.0)
Haen et al.	[56]	France	IV	PLA	20	–	–
Hamadouche et al.	[57]	France	IV	PLA	160	–	–
Haughom et al.	[58]	USA	IV	PLA	19	–	–
Hernigou et al.	[60]	France	III	PLA	85	–	–
Jorgensen et al.	[62]	Denmark	I	PLA	59	63.5	32 (54.2)
Kumar et al.	[63]	India	II	PLA	15	45.3 ± 10.5	9 (37.5)
Laurendon et al.	[65]	France	II	PLA	100	71.8 ± 11.9	43 (46.2)
Londhe et al.	[66]	India	III	PLA	204	42.5 ± 5.3	44 (21.6)
Luthra et al.	[67]	Oman	IV	PLA	18	–	–
Maisongrosse et al. ^‡^	[68]	France	III	PLA	38	–	–
Massin et al.	[70]	France	IV	PLA	2601	72 ± 9	1429 (59.3)
Moon et al.	[71]	South Korea	III	PLA	48	–	–
Nam et al.	[72]	USA	II	PLA	43	52.6 ± 6.5	13 (30.2)
Neri et al.	[73]	France	IV	PLA	212	53	70 (40.2)
Paderni et al.	[75]	Italy	IV	PLA	28	–	–
Pattyn et al.	[76]	Belgium	IV	PLA	68	–	–
Philippot et al.	[77]	France	IV	PLA	106	56	47 (52.2)
Prudhon et al.	[78]	France	III	PLA	445	–	–
Rowan et al.	[81]	USA	III	PLA	136	48.5 ± 7.2	74 (63.2)
Sanders et al.	[82]	Netherlands	IV	PLA	9	54.8 ± 5.9	6 (85.7)
Schneider et al.	[83]	France	IV	PLA	320	–	–
Singh et al. ^†^	[84]	USA	III	PLA	440	63.5 ± 12.6	307 (69.8)
Vermersch et al.	[86]	France	IV	PLA	104	73 ± 11	60 (60.0)
Vigdorchik et al.	[88]	USA	IV	PLA	485	67	207 (46.0)
Viricel et al.	[89]	France	IV	PLA	85	–	–
Yang et al.	[90]	USA	IV	PLA	150	–	–
*Combined*							
Boyer et al.	[10]	France	IV	PLA DLA	199 41	55.7 ± 11.8	112 (46.7)
Chouteau et al.	[40]	Switzerland	IV	PLA ALA	169 71	77.4 ± 5.6	147 (61.3)
Dhawan et al.	[43]	USA	II	PLA DAA	187 42	76	145 (63.9)
Ferreira et al.	[49]	France	IV	PLA DLA	465 88	71.2	338 (61.2)
Foissey et al.	[52]	France	IV	DAA PLA	98 72	72.2 ± 8.0	120 (70.6)
Martz et al.	[69]	France	IV	PLA DLA	34 6	44 ± 4	8 (25.8)
Nessler et al.	[74]	USA	IV	PLA DLA DAA	63 15 11	66	56 (60.2)
Puch et al. ^‡^	[79]	France	III	PLA DLA	86 33	49.9	35 (33.3)

Sex is reported as *n* (%). Age is reported as mean or mean ± standard deviation unless indicated otherwise

*Median is reported instead of mean

^†^Multi-armed study including patients who received ≥ 2 approaches

^‡^Single extractable cohort from a study

– Not available, *ALA*—Anterolateral approach, *DAA*—Direct anterior approach, *DLA*—Direct lateral approach, *PLA*—Posterolateral approach

**Table 2:** Patient clinical characteristics

Author	References	Approach	Indication			
			OA	Hip dysplasia	Osteonecrosis	PTA
*Anterolateral*						
Chughtai et al.	[41]	ALA	–	–	–	–
Fessy et al.^†^	[50]	ALA	–	–	–	–
Lamo-Espinosa et al.	[64]	ALA	–	–	–	–
Vielpeau et al.	[87]	ALA	508 (76.0)	–	–	–
*Direct Anterior*						
Baker et al.	[32]	DAA	–	–	–	–
Batailler et al.^†^	[33]	DAA	166 (82.6)	16 (8.0)	4 (2.0)	13 (6.5)
Homma et al.	[61]	DAA	81 (100.0)	0 (0.0)	0 (0.0)	0 (0.0)
Randelli et al.	[80]	DAA	–	–	–	–
Singh et al. ^†^	[84]	DAA	–	–	–	–
*Direct Lateral*						
Henawy and Badie	[59]	DLA	2 (8.4)	2 (8.4)	4 (16.7)	0 (0.0)
*Posterior*						
Acker	[29]	PLA	–	–	–	–
Almeida	[30]	PLA	14 (36.8)	2 (5.3)	12 (31.6)	0 (0.0)
Asselineau et al.	[31]	PLA	258 (73.7)	–	39 (11.1)	–
Assi et al.	[2]	PLA	15 (17.6)	31 (41.3)	20 (26.7)	0 (0.0)
Batailler et al.^†^	[33]	PLA	88 (87.1)	3 (3.0)	4 (4.0)	2 (2.0)
Bauchu et al.	[34]	PLA	131 (87.3)	–	13 (8.7)	–
Beckert et al.	[35]	PLA	358 (98.1)	0 (0.0)	3 (0.8)	0 (0.0)
Belgaïd et al.	[36]	PLA	121 (94.5)	2 (1.6)	1 (0.8)	–
Bouchet et al.	[37]	PLA	95 (90.5)	0 (0.0)	3 (2.9)	4 (3.8)
Chalmers et al.	[38]	PLA	–	–	–	–
Chalmers et al.	[39]	PLA	–	–	–	–
Dagneaux et al.	[42]	PLA	37 (90.2)	–	1 (2.4)	–
Dubin et al.	[44]	PLA	–	–	–	–
Dubin et al.	[45]	PLA	–	–	–	–
Dubin et al.	[46]	PLA	136 (95.8)	1 (0.7)	4 (2.8)	2 (1.4)
Epinette et al.	[47]	PLA	354 (81.0)	–	26 (5.9)	18 (4.1)
Epinette et al.	[48]	PLA	–	–	–	–
Fessy et al.^†^	[50]	PLA	–	–	–	–
Fiquet and Noyer	[51]	PLA	450 (75.0)	–	–	–
Fresard et al.	[53]	PLA	108 (85.0)	0 (0.0)	6 (4.7)	13 (10.2)
Gaillard et al.	[54]	PLA	131 (94.9)	5 (3.6)	0 (0.0)	2 (1.4)
Gkiatas et al.	[55]	PLA	–	–	–	–
Haen et al.	[56]	PLA	20 (30.3)	–	–	–
Hamadouche et al.	[57]	PLA	128 (76.2)	2 (1.2)	25 (14.9)	3 (1.8)
Haughom et al.	[58]	PLA	17 (70.1)	–	2 (8.3)	–
Hernigou et al.	[60]	PLA	–	–	–	–
Jorgensen et al.	[62]	PLA	60 (100)	0 (0.0)	0 (0.0)	0 (0.0)
Kumar et al.	[63]	PLA	–	–	1 (4.2)	5 (20.8)
Laurendon et al.	[65]	PLA	72 (72.0)	–	16 (16.0)	4 (4.0)
Londhe et al.	[66]	PLA	0 (0.0)	0 (0.0)	204 (100)	0 (0.0)
Luthra et al.	[67]	PLA	17 (56.7)	0 (0.0)	1 (3.3)	–
Maisongrosse et al. ^‡^	[68]	PLA	32 (84.2)	–	4 (10.5)	–
Massin et al.	[70]	PLA	2601 (100.0)	0 (0.0)	0 (0.0)	0 (0.0)
Moon et al.	[71]	PLA	28 (44.4)	0 (0.0)	17 (27.0)	1 (1.6)
Nam et al.	[72]	PLA	–	–	–	–
Neri et al.	[73]	PLA	164 (77.4)	23 (10.8)	25 (11.8)	6 (2.8)
Paderni et al.	[75]	PLA	26 (92.9)		–	
Pattyn et al.	[76]	PLA	55 (52.8)	9 (8.6)	2 (1.9)	–
Philippot et al.	[77]	PLA	84 (79.2)	16 (15.1)	6 (5.7)	–
Prudhon et al.	[78]	PLA	412 (96.7)	0 (0.0)	14 (3.3)	8 (1.9)
Rowan et al.	[81]	PLA	106 (77.9)	11 (8.1)	10 (7.4)	3 (2.2)
Sanders et al.	[82]	PLA	9 (90.0)	0 (0.0)	0 (0.0)	0 (0.0)
Schneider et al.	[83]	PLA	299 (90.1)	3 (0.9)	17 (5.1)	1 (0.3)
Singh et al. ^†^	[84]	PLA	–	–	–	–
Vermersch et al.	[86]	PLA	66 (63.5)	4 (3.8)	2 (1.9)	2 (1.9)
Vigdorchik et al.	[88]	PLA	–	–	–	–
Viricel et al.	[89]	PLA	29 (36.7)	9 (11.4)	37 (46.8)	–
Yang et al.	[90]	PLA	137 (89.5)	1 (0.7)	6 (3.9)	4 (2.6)
*Combined*						
Boyer et al.	[10]	PLA DLA	161 (67.1)	29 (12.1)	27 (11.3)	9 (3.8)
Chouteau et al.	[40]	PLA ALA	207 (86.3)	–	18 (7.5)	–
Dhawan et al.	[43]	PLA DAA	–	–	–	–
Ferreira et al.	[49]	PLA DLA	483 (87.3)	0 (0.0)	36 (6.5)	0 (0.0)
Foissey et al.	[52]	DAA PLA	150 (88.2)	1 (0.6)	9 (5.3)	8 (4.7)
Martz et al.	[69]	PLA DLA	0 (0.0)	0 (0.0)	40 (100)	0 (0.0)
Nessler et al.	[74]	PLA DLA DAA	–	–	–	–
Puch et al. ^‡^	[79]	PLA DLA	65 (54.6)	30 (25.2)	15 (12.6)	0 (0.0)

Data are reported as n (%) unless indicated otherwise

^†^Multi–armed study including patients who received both anterior-based and posterior-based approaches

^‡^Single extractable cohort from a study

– Not available, *ALA*—Anterolateral approach, *DAA*—Direct anterior approach, *DLA*—Direct lateral approach, *PLA*—Posterolateral approach, *OA*—osteoarthritis, *PTA*—post-traumatic arthritis

### Rate of Dislocation

Mean follow-up time varied widely across studies, ranging from 6 months [42] to 25 years [73]. Four studies reported only a minimum follow-up, which ranged from ≥ 1.5 months [58] to ≥ 3 years [49]. Dislocation rates were low regardless of approach or length of follow-up. Rates ranged from 0 to 0.9% for the ALA, 0–1.8% for the DAA, 0% for the DLA, and 0–4.7% for the PLA. Zero dislocations were reported in 4 of 5 ALA studies (80%), 7 of 8 DAA studies (87.5%), 6 of 6 DLA studies (100%), and 46 of 56 PLA studies (82.1%) (Table 3). The two studies with dislocations following DAA and ALA reported rates of 1.8% and 0.9%, respectively [84, 87]. The highest dislocation rate for PLA was 4.7% [60].

**Table 3:** Hip dislocations by intervention type

Author	References	Approach	Follow-Up (months)	Dislocation
*Anterolateral*				
Chughtai et al.	[41]	ALA	36	0 (0.0)
Fessy et al.^†^	[50]	ALA	104.4	0 (0.0)
Lamo-Espinosa et al.	[64]	ALA	49.0	0 (0.0)
Vielpeau et al.	[87]	ALA	151.1	5 (0.9)
*Direct Anterior*				
Baker et al.	[32]	DAA	81.2	0 (0.0)
Batailler et al.^†^	[33]	DAA	14	0 (0.0)
Homma et al.	[61]	DAA	15.7	0 (0.0)
Randelli et al.	[80]	DAA	23.6	0 (0.0)
Singh et al. ^†^	[84]	DAA	≥ 12	1 (1.8)
*Direct Lateral*				
Henawy and Badie	[59]	DLA	≥ 12	0 (0.0)
*Posterior*				
Acker et al.	[29]	PLA	67.2	0 (0.0)
Almeida	[30]	PLA	24–74	0 (0.0)
Asselineau et al.	[31]	PLA	57	2 (0.6)
Assi et al.	[2]	PLA	59.9	0 (0.0)
Batailler et al.^†^	[33]	PLA	14	0 (0.0)
Bauchu et al.	[34]	PLA	74.4	0 (0.0)
Beckert et al.	[35]	PLA	49	0 (0.0)
Belgaïd et al.	[36]	PLA	96	0 (0.0)
Bouchet et al.	[37]1	PLA	28	0 (0.0)
Chalmers et al.	[38]	PLA	24	0 (0.0)
Chalmers et al.	[39]	PLA	36	0 (0.0)
Dagneaux et al.	[42]	PLA	6	12 (1.1)
Dubin et al.	[44]	PLA	68.4	0 (0.0)
Dubin et al.	[45]	PLA	25.4	0 (0.0)
Dubin et al.	[46]	PLA	34.8	0 (0.0)
Epinette et al.	[47]	PLA	48	0 (0.0)
Epinette et al.	[48]	PLA	79.2	1 (3.2)
Fessy et al.^†^	[50]	PLA	104.4	0 (0.0)
Fiquet and Noyer	[51]	PLA	< 36	1 (0.2)
Fresard et al.	[53]	PLA	64.8	0 (0.0)
Gaillard et al.	[54]	PLA	152.4	0 (0.0)
Gkiatas et al.	[55]	PLA	93.6	0 (0.0)
Haen et al.	[56]	PLA	50.4	0 (0.0)
Hamadouche et al.	[57]	PLA	72	3 (1.9)
Haughom et al.	[58]	PLA	≥ 1.5	0 (0.0)
Hernigou et al.	[60]	PLA	216	4 (4.7)
Jorgensen et al.	[62]	PLA	72	1 (1.7)
Kumar et al.	[63]	PLA	12*	0 (0.0)
Laurendon et al.	[65]	PLA	120	0 (0.0)
Londhe et al.	[66]	PLA	67.5	0 (0.0)
Luthra et al.	[67]	PLA	60	0 (0.0)
Maisongrosse et al. ^‡^	[68]	PLA	54	0 (0.0)
Massin et al.	[70]	PLA	92.4	11 (0.4)
Moon et al.	[71]	PLA	37.2	0 (0.0)
Nam et al.	[72]	PLA	24	0 (0.0)
Neri et al.	[73]	PLA	303.6	0 (0.0)
Paderni et al.	[75]	PLA	27	0 (0.0)
Pattyn et al.	[76]	PLA	43.2	2 (2.9)
Philippot et al.	[77]	PLA	120	0 (0.0)
Prudhon et al.	[78]	PLA	163.3	3 (0.7)
Rowan et al.	[81]	PLA	40	0 (0.0)
Sanders et al.	[82]	PLA	38.2	0 (0.0)
Schneider et al.	[83]	PLA	34.1	0 (0.0)
Singh et al. ^†^	[84]	PLA	≥ 12	3 (0.7)
Vermersch et al.	[86]	PLA	72	0 (0.0)
Vigdorchik et al.	[88]	PLA	36	0 (0.0)
Viricel et al.	[89]	PLA	117.6	0 (0.0)
Yang et al.	[90]	PLA	61.2	0 (0.0)
*Combined*				
Boyer et al.	[10]	PLA DLA	264*	0 (0.0)
Chouteau et al.	[40]	PLA ALA	100.8	0 (0.0)
Dhawan et al.	[43]	PLA DAA	14	0 (0.0)
Ferreira et al.	[49]	PLA DLA	≥ 36	0 (0.0)
Foissey et al.	[52]	DAA PLA	70	0 (0.0)
Martz et al.	[69]	PLA DLA	116.8	0 (0.0)
Nessler et al.	[74]	PLA DLA DAA	32.2	0 (0.0)
Puch et al. ^‡^	[79]	PLA DLA	132	0 (0.0)

Dislocation is reported as *n* (%)

*Median is reported instead of mean

^†^Multi-armed study including ≥ 2 approaches

^‡^Single extractable cohort from a study

*ALA*—Anterolateral approach, *DAA*—Direct anterior approach, *DLA*—Direct lateral approach, *PLA*—posterolateral approach

### Secondary outcomes

Postoperative infection rates were similarly low across approach types. The highest reported infection rate was 2.9% for the ALA [64], 1.3% for the DAA [32], 4.2% for the DLA [59], and 5.5% for the PLA [89]. Mortality rates ranged widely across studies. One ALA study reported mortality, which was 37.1% at 12.6 years [87]. Three DAA studies reported mortality rates ranging from 1.1% and 1.2% at 14 and 15 months, respectively [33, 61], to 4.2% at 81.2 months [32]. Mortality rates for the PLA ranged from 0.0% at 38.2 months [82] to 43.7% at 25.3 years [73].

HHS scores were reported in 40.0% of studies, and PMA scores were reported in 22.2%. When both pre- and postoperative values were reported, HHS and PMA scores improved considerably for all approaches. More details on secondary outcomes are shown in Table 4.

**Table 4:** Secondary outcomes by intervention type

Author	References	Approach	Follow-Up (months)	Mortality	Infection	HHS		PMA	
						Baseline	Outcome	Baseline	Outcome
*Anterolateral*									
Chughtai et al.	[41]	ALA	36	–	2 (0.5)	51	94 ± 6	–	–
Fessy et al.^†^	[50]	ALA	104.4	–	–	–	–	–	–
Lamo-Espinosa et al.	[64]	ALA	49.0	–	2 (2.9)	–	–	10.31	15.61
Vielpeau et al.	[87]	ALA	151.1	182 (37.1)	–	–	–	–	–
*Direct Anterior*									
Baker et al.	[32]	DAA	81.2	6 (4.2)	2 (1.3)	–	–	–	–
Batailler et al.^†^	[33]	DAA	14	2 (1.1)	0 (0.0)	50.2 ± 14	95.9	–	17.3
Homma et al.	[61]	DAA	15.7	1 (1.2)	1 (1.2)	–	–	–	–
Randelli et al.	[80]	DAA	23.6	0 (0.0)	0 (0.0)	–	–	–	–
Singh et al. ^†^	[84]	DAA	≥ 12	–	–	–	–	–	–
*Direct Lateral*									
Henawy and Badie	[59]	DLA	≥ 12	0 (0.0)	1 (4.2)	36	94	8	17
*Posterior*									
Acker et al.	[29]	PLA	67.2	–	–	–	–	–	–
Almeida	[30]	PLA	24–74	–	–	–	–	–	–
Asselineau et al.	[31]	PLA	57	–	–	–	–	–	–
Assi et al.	[2]	PLA	59.9	–	1 (1.3)	–	–	–	–
Batailler et al.^†^	[33]	PLA	14	1 (1.1)	0 (0.0)	–	89.6	–	16.5
Bauchu et al.	[34]	PLA	74.4	20 (13.3)	1 (0.7)	–	–	8.9 ± 2.3	17.1 ± 1.2
Beckert et al.	[35]	PLA	49	–	–	–	–	–	–
Belgaïd et al.	[36]	PLA	96	48 (40.0)	2 (1.6)	47.9 ± 12.8	83 ± 14	–	–
Bouchet et al.	[37]1	PLA	28	–	–	–	–	–	–
Chalmers et al.	[38]	PLA	24	0 (0.0)	–	–	–	–	–
Chalmers et al.	[39]	PLA	36	0 (0.0)	2 (2.3)	–	–	–	–
Dagneaux et al.	[42]	PLA	6	–	–	–	–	–	–
Dubin et al.	[44]	PLA	68.4	–	–	–	78.95 ± 18.65	–	–
Dubin et al.	[45]	PLA	25.4	8 (1.2)	–	54.03 ± 12.94	91.44 ± 12.76	–	–
Dubin et al.	[46]	PLA	34.8	–	–	–	–	–	–
Epinette et al.	[47]	PLA	48	20 (4.8)	1 (0.2)	36.16	93.38	–	16.71
Epinette et al.	[48]	PLA	79.2	1 (3.2)	0 (0.0)	–	–	–	–
Fessy et al.^†^	[50]	PLA	104.4	–	–	–	–	–	–
Fiquet and Noyer	[51]	PLA	< 36	–	–	–	–	–	–
Fresard et al.	[53]	PLA	64.8	24 (17.9)	1 (0.7)	51.3 ± 14	88 ± 12	8 ± 3	16.3 ± 2.9
Gaillard et al.	[54]	PLA	152.4	–	0 (0.0)	45.7	94.9	11.7	17.6
Gkiatas et al.	[55]	PLA	93.6	–	2 (1.0)	–	–	–	–
Haen et al.	[56]	PLA	50.4	–	–	–	–	–	–
Hamadouche et al.	[57]	PLA	72	–	–	–	–	–	–
Haughom et al.	[58]	PLA	≥ 1.5	–	–	–	–	–	–
Hernigou et al.	[60]	PLA	216	35 (8.1)	–	–	––	–	–
Jorgensen et al.	[62]	PLA	72	9 (15.3)	1 (1.7)	56.5	89.5	–	–
Kumar et al.	[63]	PLA	12*	–	–	68.0 ± 5.3	83.0 ± 3.7	–	–
Laurendon et al.	[65]	PLA	120	19 (20.4)	–	56 ± 15.2	93 ± 8.4	11.8 ± 2.1	17 ± 1.6
Londhe et al.	[66]	PLA	67.5						
Luthra et al.	[67]	PLA	60	–	–	–	–	–	–
Maisongrosse et al. ^‡^	[68]	PLA	54.0	–	–	–	–	–	–
Massin et al.	[70]	PLA	92.4	436 (18.1)	24 (0.9)	–	–	–	–
Moon et al.	[71]	PLA	37.2	0 (0.0)	0 (0.0)	–	90.5 ± 9.8	–	–
Nam et al.	[72]	PLA	24	–	–	54.1 ± 20.5	91.2 ± 10.8	–	–
Neri et al.	[73]	PLA	303.6	76 (43.7)	2 (0.9)	51.1	83.6	11.2	16.9
Paderni et al.	[75]	PLA	27	–	–	–	–	–	–
Pattyn et al.	[76]	PLA	43.2	17 (16.3)	1 (1.0)	–	93	–	–
Philippot et al.	[77]	PLA	120	12 (13.3)	2 (1.9)	–	–	7.1 ± 0.4	15.8 ± 0.8
Prudhon et al.	[78]	PLA	163.3	92 (21.6)	2 (0.4)	–	–	–	–
Rowan et al.	[81]	PLA	40	–	–	47.7 ± 14.4	87.2 ± 16.6	–	–
Sanders et al.	[82]	PLA	38.2	0 (0.0)	0 (0.0)	–	–	–	–
Schneider et al.	[83]	PLA	34.1	–	–	–	–	–	–
Singh et al. ^†^	[84]	PLA	≥ 12	–	–	–	–	–	–
Vermersch et al.	[86]	PLA	72	12 (12.0)	1 (1.0)	56	94	13	17
Vigdorchik et al.	[88]	PLA	36	4 (0.9)	0 (0.0)	41	86	–	–
Viricel et al.	[89]	PLA	117.6	5 (6.3)	5 (5.5)	57.9 ± 13.7	95 ± 15.33	11.09 ± 2.59	17.33 ± 1.07
Yang et al.	[90]	PLA	61.2	2 (1.3)	–	42.6	82.2	–	–
*Combined*									
Boyer et al.	[10]	PLA DLA	264*	–	–	–	–	–	–
Chouteau et al.	[40]	PLA ALA	100.8	50 (22.5)	4 (1.7)	41.7 ± 13.1	83.6 ± 13.2	–	–
Dhawan et al.	[43]	PLA DAA	14	1 (0.4)	1 (0.4)	–	–	–	–
Ferreira et al.	[49]	PLA DLA	≥ 36	–	–	–	–	–	
Foissey et al.	[52]	DAA PLA	70	–	2 (1.2)	48.3 ± 6.0	83.6 ± 13.2	–	–
Martz et al.	[69]	PLA DLA	116.8	11 (35.5)	1 (2.5)	50.8 ± 15.5	95.7 ± 6.9	11 ± 3.3	17.4 ± 1.12
Nessler et al.	[74]	PLA DLA DAA	32.2	–	0 (0.0)	–	–	–	–
Puch et al. ^‡^	[79]	PLA DLA	132	4 (3.3)	1 (0.8)	39.5	98.0	8.8	17.0

Data are reported as *n*(%), mean, or mean ± standard deviation, unless indicated otherwise

^*^Median is reported instead of mean

^†^Multi-armed study including patients who received both anterior-based and posterior-based approaches

^‡^Single extractable cohort from a study

– Not available, *ALA*—Anterolateral approach, *DAA*—Direct anterior approach, *DLA*—Direct lateral approach, *PLA*—Posterolateral approach, *PMA*—Postel-Merle d’Aubigné, *HHS*—Harris Hip Score

## Discussion

This systematic review found that the use of dual mobility implants during primary THA results in low rates of postoperative hip dislocation regardless of surgical approach or length of follow-up. Sixty-three of the 75 cohorts (84.0%) reported zero dislocations at latest follow-up, demonstrating high rates of procedural success. When reported, infection rates were similarly low across all approaches, and functional hip scores (HHS and PMA) universally showed improvement from baseline to final follow-up. Importantly, this review highlights the limited available literature that report outcomes associated with the ALA, DAA, and DLA compared to the PLA. While this imbalance is expected given the historic popularity of PLA, this systematic review underscores the need for additional studies directly comparing ALA, DAA, DLA, and PLA with dual mobility implants to provide evidence for why they are not utilized more frequently for primary THA. Dual mobility implants have shown variable success in preventing dislocation in high-risk patients [91], but early recommendations to reserve dual mobility implants for patients at high risk of dislocation and salvage procedures were established from research on the first models of dual mobility cups. These implants have improved significantly since this early research to the point that their indications should be reevaluated with a fresh perspective [92, 93].

While several other systematic reviews and meta-analyses have analyzed surgical approaches in THA, the present review is the first to specifically investigate whether surgical approach in THA using dual mobility constructs affect clinical outcomes in patients undergoing primary THA. As in our current study, previous reviews have found that postoperative complications and dislocation rates are generally low following THA in non-high-risk populations, and that utilizing the DAA, ALA, DLA, or PLA may result in similar long-term outcomes [6, 94–97]. For example, a 2017 systematic review of 42 studies found that functional hip outcomes (e.g., HHS) were significantly improved for DAA in the short-term (6 weeks postoperatively), but differences were not generally sustained past that point [6]. Similarly, a meta-analysis of nine randomized controlled trials (RCTs), including 377 hips operated on via DAA and 377 via PLA, found that DAA patients had significantly reduced pain scores ≤ 72 h postoperatively, but complications and dislocation rates were low among all groups at one-year follow-up [95]. The current review contributes to this discussion with evidence that long-term complications such as dislocation and infection are similar between all approaches following THA with dual mobility implants in primary THA for low-risk populations.

While many studies have reported similar rates of long-term complications, debate still exists on the potential short-term benefits of different approaches to THA patients. For example, one RCT comparing 28 DAA and 27 PLA patients reported that functional outcomes were better after DAA in the first 3 months postoperatively (HHS 76.7 vs. 68.7; *p* = 0.08) [8], while another study compared 35 DAA and 37 PLA patients and found that functional outcomes favored DAA at 6 weeks postoperatively [98]. In contrast, a study of 20 patients undergoing bilateral, same-day THA (one hip with DAA, the other with PLA), found HHS and other functional scores were not significantly improved for either approach at 6 weeks, 3 months, 6 months, or 12 months postoperatively [99]. Similarly, an RCT of 50 ALA and 50 PLA patients reported that functional outcomes did not differ at 3-month follow-up [100]. Given these contradictory findings, additional research is needed to determine the short-term benefits of different surgical approaches following THA.

Researchers agree that approaches like the DAA involve a learning curve for surgeons, as evidenced by surgeons with less experience having longer operative times [6, 8, 97, 99–101] and higher rates of perioperative complications [33]. Fortunately, this learning curve reduces with experience and the risk of revision normalizes after as few as 20 procedures [102, 103]. This suggests that experienced surgeons at high volume centers likely have better outcomes. Additionally, multiple studies have affirmed that introducing dual mobility constructs with DAA THA presents no further risk to patients or difficulty for surgeons [33, 101], with some authors particularly optimistic that this “combination” intervention may minimize dislocations [104]. In light of conflicting evidence around short-term benefits, researchers have proposed differing policies on whether and when to implement anterior approaches for THA. Batailler, et al. suggest that DAA THA with dual mobility cups may be particularly advantageous for populations at high risk for postoperative dislocation [33]. Likewise, Thürig et al. favor DAA for elderly patients due to the potential for earlier recovery and weight-bearing [105]. Others state that the approach taken for THA should simply be based on surgeon preference and experience [98, 100]. As the debate continues, additional research is warranted to inform policies and ensure optimal treatment for different patient populations.

High-risk dislocation populations have increased risk of complications including PJI, revision, mortality, and dislocation. Risk factors for PJI include younger age, AVN, FNF, smoking, ASA ≥ 2, and diabetes. While one study by Viricel et al. [89] described an infection rate of 5.5% with 91 PLA THA, 68% of their cohort was considered high-risk for dislocation, including younger patients (mean age = 44), 60% smokers, and 25% with alcoholism. Our paper does not suggest PJI correlation with dual mobility and PLA as the above cohort inclusion criteria had known risk factors for PJI, yet notably this study had zero dislocations, which supports use of dual mobility in high-risk populations.

A residual question that remains is dual mobility cost effective in all patients. A large French registry of 80,405 patients demonstrated 0.4 relative risk of dislocation with dual mobility compared to conventional fixed bearing [106]. They looked at the cost of subsequent dislocations, revisions, and rehabilitation-unit admissions concluding through Markov analysis that the routine use of dual mobility would save 28.3 million Euros per 100,000 THA performed. Additionally, Barlow et al.[107] noted in 2013 that the average dual mobility cost $435 dollars more than fixed bearing. They also performed a Markov analysis demonstrating that the dual mobility implant was cost saving even at a rate of $1023 more than fixed bearing and cost effective up to a rate of $5,287. However, noted that was not cost effective if the unforeseen failure was above 0.29%.

Some studies have questioned the in vivo function of the dual mobility implants [92]. There have also been concerns raised about polyethylene wear, intra-prosthetic dislocations, and corrosion between articulating liners and acetabular components. Well-designed comparison outcome studies and further cost-effective analyses are in need to determine the overall utility of routine use of dual mobility constructs [108].

### Limitations

This review has several limitations. We included cohorts of larger studies when the outcome was clearly defined by surgical approach and surgical indication for dual mobility THA. Studies were excluded if they described dislocations that could not be attributed to a specific approach or preoperative diagnosis (e.g., studies that included patients with both primary osteoarthritis and femoral neck fractures, but only reported an overall dislocation rate). In doing so, a biased proportion of studies with zero dislocations may have been included, as studies reporting dislocations were more likely to be excluded for this reason. Additionally, included studies had widely varying lengths of follow-up, which makes it difficult to directly compare dislocation rates and other outcomes. The aging patient population undergoing THA is generally expected to have a higher risk of postoperative mortality (which was observed in this review), but without statistical analysis of death as a competing risk, dislocation rates were likely disproportionately low.

Unfortunately, due to considerable heterogeneity, variable quality reporting, and lack of patient-level data, this study did not stratify outcomes by surgical indication or aggregate outcomes for inferential statistics. There was also heterogeneity in reporting outcomes per patient versus per hip, which complicates direct comparisons.

## Conclusions

Total hip arthroplasty with dual mobility implants for primary osteoarthritis results in low rates of postoperative dislocation and infection and improved HHS and PMA scores regardless of surgical approach or length of follow-up. Additional studies directly comparing DAA, ALA, DLA, and PLA are needed to assess the relative advantages of these different interventions more effectively.

## Supplementary Information

**Additional file 1: Table S1.** Risk of bias assessment.

## Data Availability

All data were contained in the text and charts of published articles.

## Abbreviations

THA: Total hip arthroplasty
OA: Osteoarthritis
DAA: Direct anterior approach
ALA: Anterolateral approach
DLA: Direct lateral approach
PLA: Posterior/posterolateral approach
HHS: Harris Hip Score
PMA: Postel-Merle d’Aubigné
RCT: Randomized controlled trial
