# Supplementary material for: Comparing dislocation rates by approach following elective primary dual mobility total hip arthroplasty: a systematic review
Source: J Orthop Surg Res. 2023 Mar 22;18:226. doi: 10.1186/s13018-023-03724-6 (PMC10032016; doi:10.1186/s13018-023-03724-6)
Supplement: Supplementary file 1 — Additional file 1: Table S1. Risk of bias assessment. [file 13018_2023_3724_MOESM1_ESM.docx]

**Supplementary Table 1.** Risk of bias assessment.

| **Author** | **Year** | **Study Type*** | **Q1** | **Q2** | **Q3** | **Q4** | **Q5** | **Q6** | **Q7** | **Q8** | **Q9** | **Q10** | **Q11** | **Overall Risk of Bias** |
| --- | --- | --- | --- | --- | --- | --- | --- | --- | --- | --- | --- | --- | --- | --- |
| Acker et al. | 2017 | Case Series | Yes | Yes | Yes | Yes | No | No | Yes | Yes | No | Yes | - | Moderate |
| Almeida | 2020 | Case Series | No | Yes | Yes | Yes | Yes | Yes | Yes | Yes | No | Yes | - | Moderate |
| Asselineau et al. | 2007 | Case Series | Yes | Yes | Yes | Yes | No | Yes | Yes | Yes | No | Yes | - | Low |
| Assi et al. | 2017 | Case Series | Yes | Yes | Yes | Yes | No | Yes | Yes | Yes | No | Yes | - | Low |
| Baker et al. | 2022 | Case Series | Yes | Yes | Yes | Yes | No | Yes | Yes | Yes | Yes | Yes | - | Low |
| Batailler et al. | 2017 | Cohort^†^ | No | Yes | Yes | Yes | Yes | Yes | Yes | Yes | Yes | NA | Unclear | Moderate |
| Bauchu et al. | 2008 | Case Series | No | Yes | Yes | Yes | No | Yes | Yes | Yes | No | Yes | - | Moderate |
| Beckert et al. | 2022 | Cohort | Yes | Yes | Yes | Yes | No | Yes | Yes | Yes | Yes | Yes | Yes | Low |
| Belgaïd et al. | 2021 | Case Series | Yes | Yes | Yes | Yes | Yes | No | No | Yes | No | Yes | - | Low |
| Bouchet et al. | 2011 | Cohort | No | Yes | Yes | No | Yes | Yes | Yes | Yes | Yes | Yes | Yes | Moderate |
| Boyer et al. | 2012 | Case Series | No | Yes | Yes | Yes | Yes | Yes | Yes | Yes | No | Yes | - | Low |
| Chalmers et al. | 2020 | Case Series | Yes | Yes | Yes | Yes | No | No | No | No | No | Yes | - | Moderate |
| Chalmers et al. | 2020 | Case Series | Yes | Yes | Yes | Yes | No | Yes | Yes | Yes | No | Yes | - | Low |
| Chouteau et al. | 2020 | Case Series | Yes | Yes | Yes | Yes | No | Yes | Yes | Yes | No | Yes | - | Low |
| Chughtai et al. | 2016 | Case Series | Yes | Yes | Yes | Yes | No | Yes | No | Yes | No | Yes | - | Low |
| Dagneaux et al. | 2019 | Case Series | Yes | Yes | Yes | Yes | No | No | No | No | No | No | - | High |
| Dhawan et al. | 2022 | Case Series | Yes | No | Yes | Yes | No | Yes | Yes | Yes | Yes | Yes | - | Moderate |
| Dubin et al. | 2020a | Case Series | Yes | Yes | Yes | Unclear | No | Yes | Yes | Yes | No | Yes | - | Low |
| Dubin et al. | 2020b | Case Series | Yes | Yes | Yes | Yes | No | No | Yes | Yes | No | Yes | - | Low |
| Dubin et al. | 2019 | Case Series | Yes | Yes | Yes | No | No | Yes | Yes | Yes | No | Unclear | - | Moderate |
| Epinette et al. | 2014 | Case Series | Yes | Yes | Yes | Yes | No | Yes | Yes | Yes | No | Unclear | - | Low |
| Epinette et al. | 2022 | Case Series | Yes | Yes | Yes | Yes | No | Yes | Yes | Yes | Yes | Yes | - | Low |
| Ferreira et al. | 2017 | Case Series | Yes | Yes | Yes | Yes | No | Yes | Yes | No | Yes | Yes |  | Low |
| Fessy et al. | 2019 | Cohort^†^ | Unclear | No | Yes | No | No | Yes | Yes | Yes | Yes | Yes | No | High |
| Fiquet & Noyer | 2006 | Case Series | Unclear | No | Yes | Yes | No | No | Yes | Yes | No | Yes | - | High |
| Foissey et al. | 2023 | Case Series | Yes | Yes | Yes | Unclear | No | Yes | Yes | Yes | Yes | Yes | - | Low |
| Fresard et al. | 2013 | Case Series | No | Yes | Yes | Yes | No | Yes | Yes | Yes | No | Yes | - | Moderate |
| Gaillard et al. | 2019 | Case Series | Yes | Yes | Yes | Yes | Yes | Yes | Yes | Yes | No | Yes | - | Low |
| Gkiatas et al. | 2022 | Case Series | Yes | Yes | Yes | Yes | No | Yes | Yes | Yes | Yes | Yes | - | Low |
| Haen et al. | 2015 | Case Series | Yes | Yes | Yes | Yes | No | Yes | Yes | Yes | No | Yes | - | Moderate |
| Hamadouche et al. | 2012 | Case Series | Yes | Yes | Yes | Unclear | No | Yes | No | Yes | No | Yes | - | Moderate |
| Haughom et al. | 2016 | Cohort | Yes | Yes | Yes | Yes | No | Yes | Yes | No | No | Yes | Yes | Moderate |
| Henawy & Badie | 2017 | Case Series | No | Yes | Yes | Yes | Yes | Yes | Yes | Yes | No | Yes | - | Low |
| Hernigou et al. | 2016 | Cohort | No | Yes | Yes | Yes | Yes | Yes | Yes | Yes | No | Yes | Yes | Low |
| Homma et al. | 2017 | Case Series | Unclear | Yes | Yes | Yes | Yes | Yes | Yes | Yes | No | Yes | - | Low |
| Jorgensen et al. | 2022 | Cohort | Yes | Yes | Yes | Yes | Yes | Yes | Yes | Yes | No | Yes | Yes | Low |
| Kumar et al. | 2022 | Case Series | Yes | Yes | Yes | Yes | Yes | Yes | No | Yes | No | Yes | - | Low |
| Lamo-Espinosa et al. | 2021 | Case Series | Yes | Yes | Yes | Unclear | Yes | Yes | Yes | Yes | No | Yes | - | Low |
| Laurendon et al. | 2018 | Case Series | Yes | Yes | Yes | Yes | Yes | Yes | Yes | Yes | Yes | Yes | - | Low |
| Londhe et al. | 2022 | Case Series | Yes | Yes | Yes | Yes | Yes | Yes | Yes | Yes | No | Yes | - | Low |
| Luthra et al. | 2016 | Case Series | Yes | Yes | Yes | Yes | Unclear | No | Yes | No | Yes | Yes | - | Moderate |
| Maisongrosse et al. | 2015 | Cohort | Yes | Yes | Yes | Yes | Yes | Yes | Yes | Yes | No | Yes | Yes | Low |
| Martz et al. | 2017 | Case Series | Yes | Yes | Yes | Unclear | Yes | Yes | Yes | Yes | No | Yes | - | Low |
| Massin et al. | 2012 | Case Series | Yes | Yes | Yes | Yes | No | Yes | Yes | Yes | No | Yes | - | Low |
| Moon et al. | 2022 | Cohort | Yes | Yes | Yes | Yes | Yes | Yes | Yes | Yes | Yes | Unclear | Yes | Low |
| Nam et al. | 2019 | Case Series | Yes | No | Unclear | Unclear | Unclear | Yes | Yes | Yes | No | Yes | - | Moderate |
| Neri et al. | 2017 | Case Series | Yes | Yes | Yes | Yes | Yes | Yes | No | Yes | No | Yes | - | Low |
| Nessler et al. | 2020 | Case Series | Yes | No | Unclear | Unclear | Unclear | Yes | Yes | Yes | No | Yes | - | Moderate |
| Paderni et al. | 2022 | Case Series | Yes | Yes | No | Unclear | Yes | Yes | Yes | Yes | Yes | Yes | - | Low |
| Pattyn et al. | 2018 | Case Series | No | Yes | No | Unclear | No | Yes | Yes | Yes | No | Yes | - | Moderate |
| Philippot et al. | 2006 | Case Series | Yes | Yes | Yes | Yes | Yes | Yes | No | Yes | Yes | No | - | Low |
| Prudhon et al. | 2017 | Case Series | No | Yes | Yes | Unclear | Unclear | Yes | Yes | Yes | No | Yes | - | Moderate |
| Puch et al. | 2017 | Cohort | Yes | Yes | Yes | Yes | No | Yes | Yes | Yes | No | Unclear | Yes | Low |
| Randelli et al. | 2020 | Case Series | Yes | No | No | Unclear | Unclear | Yes | Yes | Yes | No | Yes | - | High |
| Rowan et al. | 2017 | Case Series | Yes | Yes | Yes | Unclear | Unclear | Yes | Yes | Yes | No | Yes | - | Low |
| Sanders et al. | 2013 | Case Series | Yes | Yes | Yes | Yes | Yes | Yes | Yes | Yes | No | Yes | - | Low |
| Schneider et al. | 2021 | Case Series | No | Yes | Yes | Yes | No | Yes | Yes | Yes | No | Yes | - | Low |
| Singh et al. | 2022 | Cohort | Yes | No | Yes | Yes | Yes | Yes | Yes | Yes | Unclear | Unclear | Yes | Moderate |
| Vermersch et al. | 2015 | Case Series | Unclear | Yes | Yes | Yes | Yes | Yes | Yes | No | No | Yes | - | Low |
| Vielpeau et al. | 2011 | Case Series | No | Unclear | Yes | Yes | Yes | Yes | Yes | Yes | No | Yes | - | Low |
| Vigdorchik et al. | 2015 | Case Series | Yes | Yes | Yes | Yes | Yes | Yes | No | Yes | No | Yes | - | Low |
| Viricel et al. | 2022 | Case Series | Yes | Yes | Yes | Yes | Yes | Yes | Yes | Yes | Yes | Yes | - | Low |
| Yang et al. | 2022 | Case Series | Yes | Yes | Yes | Yes | Yes | Yes | No | Yes | No | Yes | - | Low |

*Study design determination is according to the utility of the study in the context of the current review, with the comparator groups of anterior-based vs. posterior-based surgical approaches for total hip arthroplasty.

^†^ Cohort studies have 11 questions in the Joanna Briggs Institute appraisal form, whereas case series only have 10 questions.

-, not available; NA, not applicable.

**Case Series**

Q1. Were there clear criteria for inclusion in the case series?

Q2. Was the condition measured in a standard, reliable way for all participants included in the case series?

Q3. Were valid methods used for identification of the condition for all participants included in the case series?

Q4. Did the case series have consecutive inclusion of participants?

Q5. Did the case series have complete inclusion of participants?

Q6. Was there clear reporting of the demographics of the participants in the study?

Q7. Was there clear reporting of clinical information of the participants?

Q8. Were the outcomes or follow up results of cases clearly reported?

Q9. Was there clear reporting of the presenting site(s)/clinic(s) demographic information?

Q10. Was statistical analysis appropriate?

**Cohort Study**

Q1. Were the two groups similar and recruited from the same population?

Q2. Were the exposures measured similarly to assign people to both exposed and unexposed groups?

Q3. Was the exposure measured in a valid and reliable way?

Q4. Were confounding factors identified?

Q5. Were strategies to deal with confounding factors stated?

Q6. Were the groups/participants free of the outcome at the start of the study (or at the moment of exposure)?

Q7. Were the outcomes measured in a valid and reliable way?

Q8. Was the follow up time reported and sufficient to be long enough for outcomes to occur?

Q9. Was follow up complete, and if not, were the reasons to loss to follow up described and explored?

Q10. Were strategies to address incomplete follow up utilized?

Q11. Was appropriate statistical analysis used?
